# CD274 promotes cell cycle entry of leukemia-initiating cells through JNK/Cyclin D2 signaling

**DOI:** 10.1186/s13045-016-0350-6

**Published:** 2016-11-17

**Authors:** Xia Fang, Chiqi Chen, Fangzhen Xia, Zhuo Yu, Yaping Zhang, Feifei Zhang, Hao Gu, Jiangbo Wan, Xiaocui Zhang, Wei Weng, Cheng Cheng Zhang, Guo-Qiang Chen, Aibing Liang, Li Xie, Junke Zheng

**Affiliations:** 1 Department of Hematology, Shanghai Tongji Hospital, Shanghai Tongji University School of Medicine, Shanghai, China; Hongqiao International Institute of Medicine,Shanghai Tongren Hospital, Key Laboratory of Cell Differentiation and Apoptosis of Chinese Ministry of Education, Shanghai Jiao Tong University School of Medicine, Shanghai, China; 2 Department of Hematology, Shanghai Xinhua Hospital, Shanghai Jiao Tong University School of Medicine, Shanghai, China; 3 Departments of Physiology and Developmental Biology, UT Southwestern Medical Center, Dallas, TX 75390 USA

**Keywords:** Programmed death ligand-1/CD274, Leukemia-initiating cells, JNK, Cyclin D2, Cell cycle entry

## Abstract

**Background:**

CD274 (programmed death ligand 1, also known as B7H1) is expressed in both solid tumors and hematologic malignancies and is of critical importance for the escape of tumor cells from immune surveillance by inhibiting T cell function via its receptor, programmed death 1 (PD-1). Increasing evidence indicates that functional monoclonal antibodies of CD274 may potently enhance the antitumor effect in many cancers. However, the role of CD274 in leukemia-initiating cells (LICs) remains largely unknown.

**Methods:**

We established an MLL-AF9-induced acute myeloid leukemia (AML) model with wild-type (WT) and CD274-null mice to elucidate the role of CD274 in the cell fates of LICs, including self-renewal, differentiation, cell cycle, and apoptosis. RNA sequencing was performed to reveal the potential downstream targets, the results of which were further validated both in vitro and in vivo.

**Results:**

In silico analysis indicated that CD274 level was inversely correlated with the overall survival of AML patients. In Mac-1^+^/c-Kit^+^ mouse LICs, CD274 was expressed at a much higher level than in the normal hematopoietic stem cells (HSCs). The survival of the mice with CD274-null leukemia cells was dramatically extended during the serial transplantation compared with that of their WT counterparts. CD274 deletion led to a significant decrease in LIC frequency and arrest in the G1 phase of the cell cycle. Interestingly, CD274 is not required for the maintenance of HSC pool as shown in our previous study. Mechanistically, we demonstrated that the levels of both phospho-JNK and Cyclin D2 were strikingly downregulated in CD274-null LICs. The overexpression of Cyclin D2 fully rescued the loss of function of CD274. Moreover, CD274 was directly associated with JNK and enhanced the downstream signaling to increase the Cyclin D2 level, promoting leukemia development.

**Conclusions:**

The surface immune molecule CD274 plays a critical role in the proliferation of LICs. The CD274/JNK/Cyclin D2 pathway promotes the cell cycle entry of LICs, which may serve as a novel therapeutic target for the treatment of leukemia.

**Electronic supplementary material:**

The online version of this article (doi:10.1186/s13045-016-0350-6) contains supplementary material, which is available to authorized users.

## Background

Acute myeloid leukemia (AML) is the most common type of leukemia in adults, which is considered a clonal disease with diverse biologic and cytogenetic features [1]. In the past decades, the overall survival of AML patients has been improved due to the emergence of many newly developed regimens, including chemotherapy, radiotherapy, immune therapy, and allogeneic hematopoietic stem-cell transplantation (allo-HSCT) [2]. However, a large number of AML patients still suffer from the relapse of these disorders even after receiving the combination of all different treatments [3–5]. Currently, leukemia-initiating cells (LICs) are considered to be responsible for the initiation, development, and relapse of all types of leukemia. Therefore, identification of ideal molecules to target LICs may be an efficient way for the eradication of leukemia. Our previous study indicated that an inhibitory immune receptor, leukocyte immunoglobulin-like receptor subfamily B member 2 (LILRB2), is critical for LIC maintenance and leukemia development [6, 7]. Interestingly, similar surface immune ligands and receptors, such as CD47, interleukin-3 receptor, and CD97, have also been revealed to play critical roles in the stemness maintenance or immune escape in both solid caners and malignant hematopoietic diseases [8–11]. Targeting such surface immune molecules with either monoclonal antibodies or engineered chimeric antigen receptor T cells (CAR T) may be the most promising and powerful strategy to eliminate LICs and other types of cancer stem cells [12–14].

CD274, one of the most important members of B7/CD28 family, is expressed on activated T cells, B cells, and NKT cells [15, 16]. CD274 binds PD-1 to deliver the potent inhibitory signaling that inactivates T cells or other immune cells, which is essential to the maintenance of homeostasis of the immune systems. Recently, innate and adaptive immune resistance induced by CD274 has been reported in many types of cancers. For instance, the loss of function of PTEN or activation of PI3K/AKT/mTOR signaling can enhance the expression of CD274 in tumor cells, which further leads to the escape from the surveillance by the immune system [17, 18].

Interestingly, several studies also indicated that CD274 may serve as a “receptor” to deliver “reverse” signaling [19–21]. For example, Dong et al. discovered that total PD-L1 and membrane PD-L1 protein were overexpressed in some DLBCL cells, and the AKT/mTOR pathway was activated by PD-1/Fc stimulation, which indicated that PD-1/PD-L1 directly activated the intracellular oncogenic signaling pathway in tumor cells [21]. The intracellular domain of CD274 contains 30 amino acids and may serve as a potential region to recruit other co-factors to initiate downstream signaling [22]. On the other hand, CD274 has been detected both in the cytoplasm and nucleus [23]. These results indicate CD274 may have distinct functions in these tumors other than serving as an inhibitor of immune cells. Nevertheless, how CD274 exerts this effect on LICs remains largely unknown.

Herein, we revealed that CD274 was highly expressed in AML cells and inversely correlated with the overall survival of AML patients. We further unraveled the function of CD274 in the regulation of LIC proliferation using a murine AML model. Intriguingly, CD274-null LICs manifested a severe defect in proliferation, which might have been caused by the cell cycle arrest in G1 phase both in vitro and in vivo. CD274 deletion almost completely abrogated the leukemogenesis ability of LICs and dramatically extended the survival of leukemic mice. CD274 collaborates with JNK signaling in the upregulation of Cyclin D2 to sustain the LIC pool and promote AML development.

## Methods

### Mice

C57BL/6 mice were purchased from the Shanghai SLAC Laboratory Animal Co. Ltd., China. The CD274 knockout mice with a C57BL/6 background were kindly provided by Dr. Lieping Chen from the Johns Hopkins University School of Medicine (Baltimore, MA, USA). All animals were housed under specific pathogen-free conditions at the Laboratory Animal Care-approved facility of Shanghai Jiao Tong University School of Medicine, China. All animal experimental procedures were approved according to the Guide for Central Animal Care and Use of the Committee of Shanghai Jiao Tong University School of Medicine.

### Retroviral infection and transplantation

MLL-AF9-expressing retroviruses were produced in 293T cells by co-transfection with the MSCV-MLL-AF9-IRES-YFP encoding plasmid and the pCL-ECO packaging plasmid. Then, Lin^−^ fetal liver cells were isolated and infected with MLL-AF9 retroviruses with 4 μg/mL polybrene and centrifuged at 1500 rpm for 2 h at 37 °C as previously described [24]. Cells were cultured overnight in StemSpan SFEM medium (StemSpan, USA) with 20 ng/mL SCF, 20 ng/mL IL-3, and 10 ng/mL IL-6, followed by another round of spin infection. Next, infected cells (2.5 × 10^5^) were transplanted into lethally irradiated (10 Gy) C57BL/6 mice by retro-orbital injection. Further, indicated numbers (0.2–0.4 × 10^4^) of leukemia cells from the primary leukemic mice were injected into the recipient mice for secondary transplantation.

### Flow cytometry analysis

Analyses of leukemic lineages and apoptosis were performed as described earlier [6]. Briefly, for analysis of lineages and LICs, bone marrow cells were stained with anti-mouse Mac-1-PE, anti-mouse Gr-1-APC, anti-mouse CD3e-PE, anti-mouse B220-PE, and anti-mouse c-Kit-APC monoclonal antibodies (eBioscience, USA). For detection of CD274 expression in Mac-1^+^/c-Kit^+^ LICs of murine AML model, anti-mouse CD274-biotin and streptavidin-PE (secondary antibody) were used (eBioscience, USA). Cell cycle status was determined in purified Mac-1^+^/c-Kit^+^ LICs with Pyronin Y and Hoeschst 33342 staining (Sigma, USA) as previously described [25]. Apoptosis analysis was conducted in purified Mac-1^+^/c-Kit^+^ LICs with anti-Annexin V-PE and 7-AAD staining (BD Pharmingen, USA) according to the manufacturer’s instructions.

### Quantitative RT-PCR

Mac-1^+^/c-Kit^+^ LICs of the murine AML model were sorted by flow cytometry for further RNA extraction. First-strand cDNA was transcribed using Reverse Transcriptase XL (AMV) (Takara, Japan) and allowed to react with the following primers (10 μM/L) containing 2 × ABI SYBR® Green PCR master mix to measure the expression of the studied genes in AML LICs. Details of the primer sequences used are shown in Additional file 1: Table S1.

### Western blotting and co-immunoprecipitation

A combination of plasmids of PLVX-mouse CD274-Strep II, XZ201-mouse Cyclin D2-HA, and CMV5.1-JNK-Fc were transfected into 293T cells followed by a co-immunoprecipitation (co-IP) process to further analyze their interaction. Alternatively, the CD274 or JNK overexpressed plasmids were transfected into 293T cells to observe the expression of Cyclin D2, p-JNK, and JNK. Whole cell lysates and co-IP samples were electrophoresed on 10% sodium dodecyl sulfate polyacrylamide gels and transferred onto polyvinylidene difluoride membranes (Millipore, USA). After electrophoresis and membrane transfer, the immunoblots were probed with the following primary antibodies: anti-mouse Strep II (Genescript, USA), anti-mouse HA (CST, USA), anti-phospho JNK (Abways, China), anti-JNK (Abways, China), anti-Cyclin D2 (Boster, China), and anti-β-actin (Calbiochem, USA).

### Library construction and RNA sequencing

Total RNA from 10 mg tissue was isolated with depleting genomic DNA for RNA-seq library construction following the standard TruSeq RNA sample preparation v2 protocol (Illumina). The sequencing libraries were then sequenced using the Illumina HiSeq2500 platform. From the reads averaging 50 bp in length, we generated 16.5 ± 1.3 million reads per sample. Further, we aligned the reads to the mouse reference genome (GRCm38, Ensembl build) using Tophat (version 2.0.12), yielding an average mapping rate of 90.3 ± 2.3%. Gene expression levels, which were represented as fragments per kilobase per million mapped reads (FPKM), were obtained for 27,180 genes/transcripts. The RNA-sequencing data were deposited to the Gene Expression Omnibus (GEO) repository under number GSE85193.

### Function analysis

Gene ontology enrichment analysis was carried out by the Bioconductor package “topGO,” and KEGG pathway enrichment analysis was conducted by the Bioconductor package “GSEABase” (http://www.r-project.org/; http://www.bioconductor.org/packages/release/bioc/html/GSEABase.html). Terms were accepted if they were hit more than one gene, and Fisher’s exact test *P* value was <0.05.

### Colony-forming unit and cell proliferation assays

The indicated number of wild-type (WT) and CD274-null leukemia cells were sorted and plated in methylcellulose (M3534, Stem Cell Technologies) according to the manufacturer’s protocols. The numbers of colonies were calculated after 7–10-day culture. In some cases, the lentiviral vector pLKO.1-GFP was used to express shRNAs designed to target CD274 (sequences listed in Additional file 1: Table S1). WT and CD274-null Mac-1^+^/c-Kit^+^ LICs were infected with shRNA targeting JNK and sorted by flow cytometry, then the cells were cultured both in solution or methylcellulose medium. The cell and colony numbers were calculated at indicated time points.

### Statistical analysis

Statistical analysis was performed using GraphPad and SPSS software program, version 19.0. Statistical differences between groups were determined by Student’s *t* test. The Kaplan-Meier method with log-rank test was utilized to compare survival data among groups. Results were expressed as means ± SEM. A probability level of *P* < 0.05 was regarded as statistically significant.

## Results

### CD274 is highly expressed on LICs and promotes AML development

**Fig. 1:**
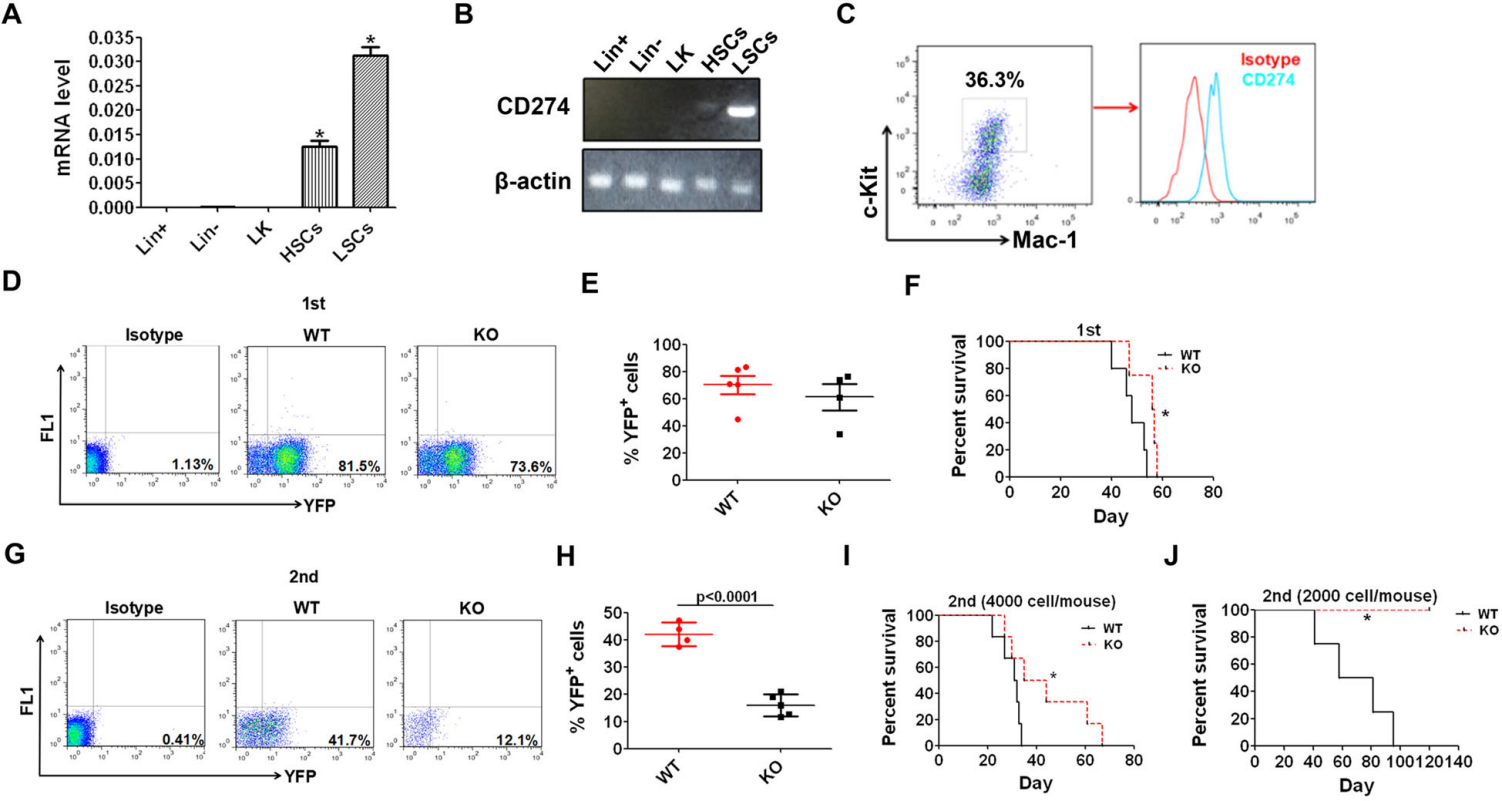
CD274 is highly expressed in LICs and promotes AML development. **a**, **b** CD274 expression levels were determined in mouse LICs, HSCs, and other hematopoietic cells (LK, Lin^−^, and Lin^+^ cells) by real-time RT-PCR or semi-quantitative PCR. **c** The expression of CD274 in mouse LICs was examined by flow cytometric analysis. **d**, **e** The frequencies of WT and CD274-null leukemia cells (YFP^+^) in the peripheral blood in primary recipient mice 3 weeks post-transplantation were analyzed. Normal mouse bone marrow cells were used as background (bg) fluorescence control. Representative flow cytometric plots (**d**) and quantitative results (**e**) are shown (*n* = 4–5). **f** MLL-AF9-induced WT and CD274-null hematopoietic stem/progenitors was transplanted into recipient mice, followed by the analysis of overall survival upon the primary transplantation (*n* = 4–5). **g**, **h** The frequencies of WT and CD274-null leukemia cells in the peripheral blood were determined three weeks after the secondary transplantation. Normal mouse bone marrow cells were used as bg fluorescence control. Representative flow cytometric plots (**g**) and quantitative results (**h**) are depicted (*n* = 4–5). **i**, **j** Representative results of the overall survival of the recipient mice receiving 4000 (**i**) or 2000 (**j**) WT or CD274-null leukemia cells upon the secondary transplantation (*n* = 6 for **i**, and *n* = 4–5 for **j**). (*p < 0.05)

To explore the role of CD274 in leukemogenesis, we first examined the expression of CD274 in phenotypic Mac-1^+^/c-Kit^+^ LICs and different cell population of normal bone marrow hematopoietic cells by quantitative RT-PCR. As illustrated in Fig. 1a, b, CD274 was expressed on LICs at a level that was higher than that in normal hematopoietic stem cells (HSCs), hematopoietic progenitors (LK, Lin^−^ cells), and differentiated cells (Lin^+^ cells). The CD274 level on LICs was further confirmed by flow cytometric analysis (Fig. 1c). Then, we investigated the function of CD274 in the AML model induced by the MLL-AF9 oncogene (tagged with yellow fluorescent protein (YFP)) as previously described [6]. Although no significant difference was observed between the frequencies of YFP^+^ leukemia cells from WT and CD274-null leukemic mice (Fig. 1d, e), the mice transplanted with MLL-AF9-induced CD274-null hematopoietic stem/progenitor cells developed leukemia somewhat more slowly than their WT counterparts (Fig. 1f, *p* < 0.05). We further performed a secondary transplantation with 4000 WT and CD274-null AML cells from primary recipients and found that the percentage of CD274-null YFP^+^ leukemia cells in the peripheral blood was notably reduced compared to that in the WT ones (42.03 ± 2.15 vs 16.02 ± 1.79, Fig. 1g, h) three weeks after transplantation. More importantly, the recipients transplanted with CD274-null AML cells had significantly extended survival (Fig. 1i, *p* < 0.05). Besides, our previous study showed that CD274 deletion had no effect on normal HSCs [26]. More strikingly, the recipients transplanted with 2000 CD274-null leukemia cells had significantly prolonged survival compared to that of their WT counterparts (Fig. 1j, *p* < 0.05).

Then, we further examined the lineages of CD274-null leukemia cells and found that the differentiation status was not altered according to the analysis with the surface markers of Mac-1 and Gr-1 (Mac-1^+^/Gr-1^+^ cells representing a more mature leukemia cell population) (Additional file 2: Figure S1A-B). This finding suggests that the delayed AML development upon CD274 deletion might not have resulted from the enhanced differentiation. In addition, exceedingly few CD3^+^ or B220^+^ (markers for lymphoid lineages) leukemia cells were observed in the recipient mice, indicating the characteristics of a myeloid leukemia model (Additional file 2: Figure S1C). Consistently, we also found that the values of the size and weight of the spleen of the recipients that received primary CD274-null leukemia cells were lower than those of the WT controls (Additional file 2: Figure S1D-F). The histological hematoxylin/eosin staining also revealed that there were much less infiltrated leukemia cells in the spleen of the mice transplanted with CD274-null leukemia cells (Additional file 2: Figure S1G).

Interestingly, the in silico analysis of data extracted from the curated databases (the HemaExplorer, http://servers.binf.ku.dk/hemaexplorer/) or Leukemia Gene Atlas (LGA) (http://www.leukemia-gene-atlas.org/LGAtlas/) showed that the level of expression of CD274 in AML cells was much higher than that in normal hematopoietic stem cells, which was inversely correlated to the overall survival of AML patients (Additional file 3: Figure S2A-B) [27]. Taken together, CD274 maintains the proliferation of LICs and may serve as a potential biomarker for AML.

### CD274 promotes cell cycle entry of LICs

**Fig. 2:**
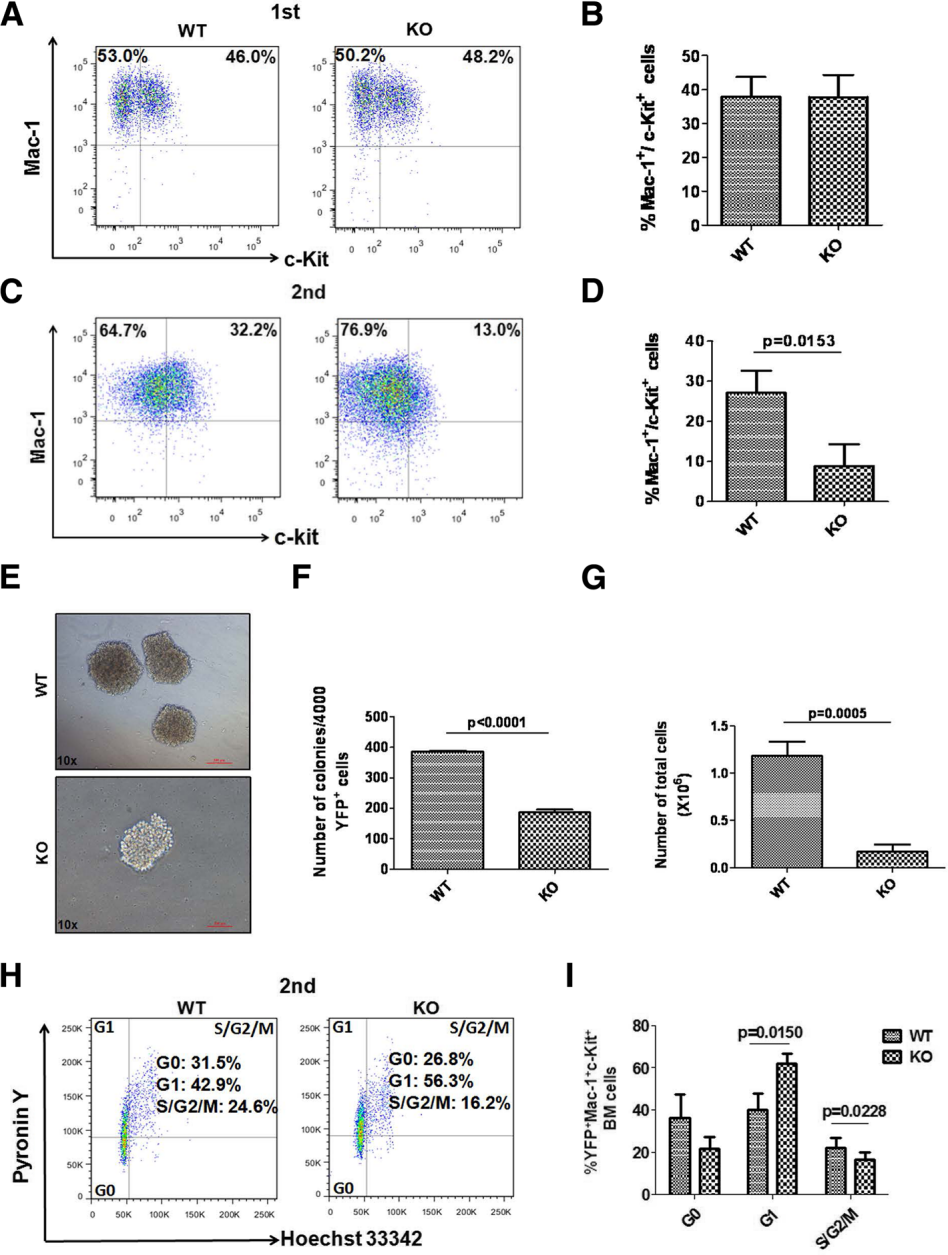
CD274 promotes cell cycle entry of LICs. **a** Representative flow cytometric plot for the percentages of Mac-1^+^/c-Kit^+^ cells (enriched for LICs) in the recipient mice upon primary transplantation. **b** Quantification results for panel **a** (*n* = 3). **c** Representative flow cytometric plot for the percentages of Mac-1^+^/c-Kit^+^ cells in the recipient mice upon secondary transplantation. **d** Quantification results for **c** (*n* = 3). **e**–**g** Representative images for the colony forming units of WT and CD274-null AML cells upon secondary transplantation. Colony numbers (**f**) and total cell number of colonies (**g**) in **e** were evaluated (*n* = 3, *right*). **h**, **i** Representative flow cytometric plots for cell cycle in WT and CD274-null Mac-1^+^/c-Kit^+^ cells (enriched for LICs) determined by Pyronin Y and Hoechst 33342 staining upon secondary transplantation (**h**). Quantitative data for panel **h** (**i**, *n* = 3)

To identify the underlying mechanisms of CD274 functions in the stemness regulation of LICs, we further examined the LIC frequency in the bone marrow of primary and secondary recipients. Although no significant difference in LIC (Mac-1^+^/c-Kit^+^ cells) frequency was found between the WT and CD274-null recipients upon the primary transplantation (Fig. 2a, b), the LIC frequency in the CD274-null recipients was considerably lower than that in the WT ones after the secondary transplantation (Fig. 2c, d). Consistently, the results of the colony-forming unit assay showed that CD274-null AML cells generated much lower colony numbers and total cell numbers than WT controls (Fig. 2e–g), indicating that CD274 depletion led to a notable reduction in the proliferation potential of LICs.

To understand how CD274 controls the growth of LICs, we determined the cell cycle status of Mac-1^+^/c-Kit^+^ LICs by Hoechst 33342 and Pyronin Y staining. Intriguingly, we found that most of the CD274-null LICs were arrested in the G1 phase unlike their WT counterparts (Fig. 2h, i). In addition, there was no significant difference between WT cells and CD274-null LICs in LIC differentiation upon the secondary transplantation (Additional file 4: Figure S3A-B). Furthermore, no distinct difference was found between the apoptosis in WT and CD274-null AML cells from either primary or secondary transplantation (Additional file 4: Figure S3C-F). These data indicate that CD274 controls the cell cycle entry to maintain the pool of LICs in the bone marrow.

### CD274 maintains the Cyclin D2 level to accelerate AML development

**Fig. 3:**
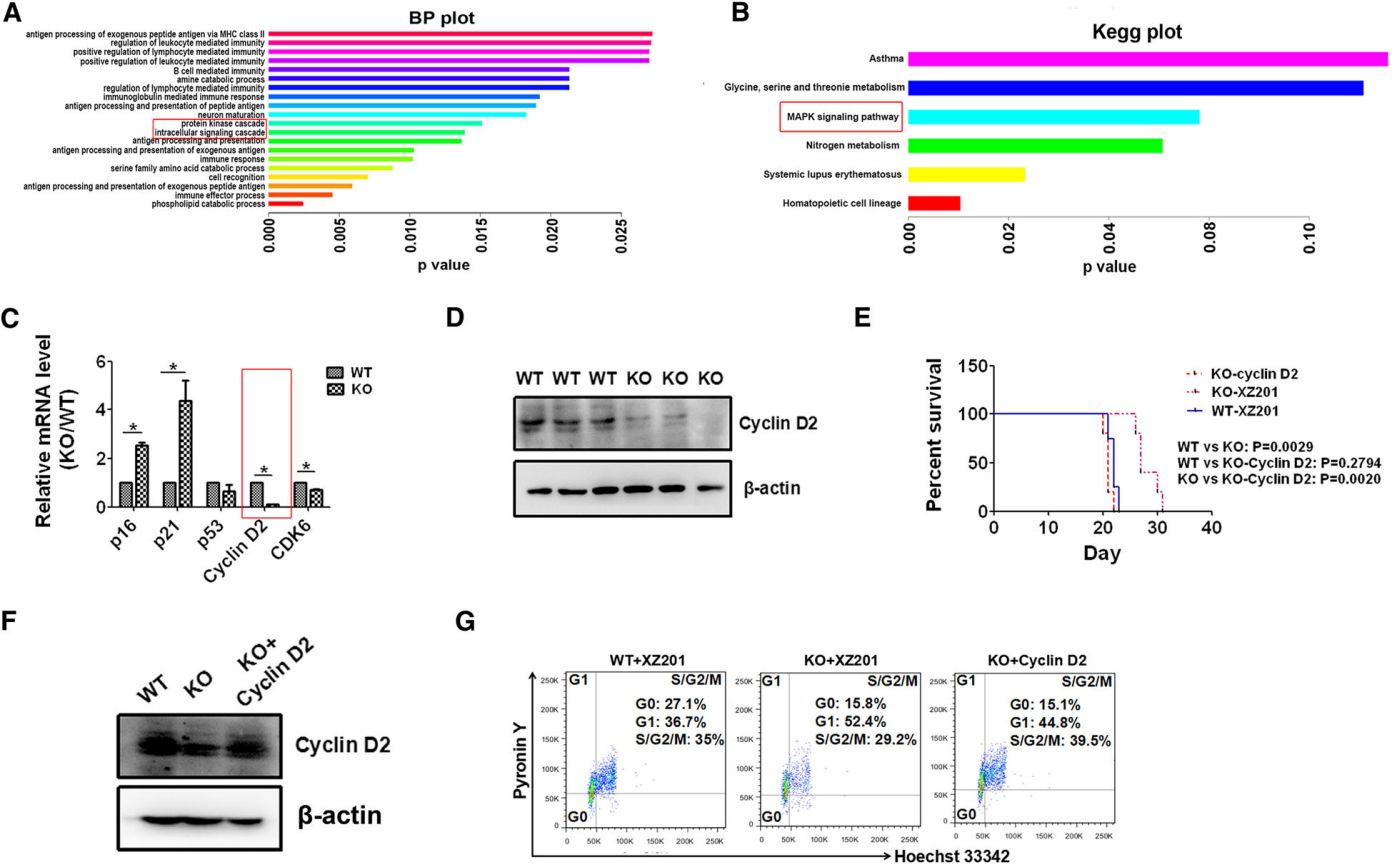
CD274 maintains the Cyclin D2 level to accelerate AML development. **a**, **b** GO and KEGG analyses of differentially expressed genes in WT and CD274-null LICs from RNA sequencing data are shown. Candidate genes involved in the biologic progress and the pathway are *highlighted*. **c** Candidate genes were further confirmed with WT and CD274-null LICs using real-time RT-PCR. **d** Cyclin D2 levels in WT and CD274-null LICs were detected by Western blotting analysis. **e** Long-rank test analysis for the overall survival of the recipient mice receiving WT AML cells, CD274-null AML cells, and Cyclin D2-overexpressed CD274-null AML cells (*n* = 4–5). **f** Cyclin D2 level was examined in WT AML cells, CD274-null AML cells, and Cyclin D2-over-expressed CD274-null AML cells in **e**. **g** The stages of cell cycle were analyzed by Pyronin Y and Hoechst 33342 staining in WT LICs, CD274-null LICs, and Cyclin D2-over-expressed CD274-null LICs

To determine the potential targets of LICs controlled by CD274, we performed RNA-sequencing with WT and CD274-null LICs. After aligning the reads to the mouse reference genome, we obtained an average mapping rate of 90.3 ± 2.3%. Gene expression levels, which were represented as FPKM, were evaluated among 27,180 genes/transcripts. Using a cutoff of *P* value <0.01 and a fold change of >1.5, a total of 457 candidate genes were characterized by comparing CD274-null with WT groups. Gene ontology analysis revealed that the differentially expressed genes were mainly involved in the biological process of many “immune-related activities” (Fig. 3a). Intriguingly, GO analysis indicated CD274 might also play a key role in the process of “protein kinase cascade” and “intracellular signaling cascade” (Fig. 3a). The KEGG analysis further revealed that these genes were enriched in the “MAPK signaling pathway” or “Hematopoietic cell lineage” (Fig. 3b). To investigate the reduced proliferation abilities and cell cycle arrest in the G1 phase in CD274-null LICs, we further analyzed a number of candidate genes related to proliferation and cell cycle activators or inhibitors (Additional file 5: Figure S4). Although the candidate genes related to proliferation were not significantly changed, several cell cycle regulators, including p16, p21, Cyclin D2, and CDK6, were markedly up- or downregulated (Additional file 5: Figure S4). The RNA-sequencing results indicated that CD274 might be involved in the AML development through the cell cycle regulation.

To confirm whether these cell cycle-related genes were potential downstream targets of CD274, we examined their expressions in WT and CD274-null LICs by quantitative RT-PCR and revealed that the levels of both p21 and p16 were increased, whereas those of Cyclin D2 and CDK6 were decreased in CD274-null LICs compared to WT ones (Fig. 3c). Since the expression of Cyclin D2, which is a key regulator in promoting G1-S transition of cell cycle [28], was most significantly downregulated (reduced to 10% of that in WT LICs) among these candidate genes (Fig. 3c), we decided to determine whether Cyclin D2 served as a downstream target for CD274 in leukemogenesis. We further confirmed that Cyclin D2 level consistently decreased in CD274-null LICs by Western blotting analysis (Fig. 3d). Cyclin D2 was then ectopically expressed in the CD274-null AML cells, followed by the transplantation into the recipient mice to test whether it could rescue the loss of function of CD274. Importantly, the recipient mice transplanted with Cyclin D2-overexpressed CD274-null AML cells developed AML much faster than the CD274-null AML counterparts, which was comparable to the case in the WT controls (Fig. 3e, f). Meanwhile, the blockage of G1 to S phase transition in CD274-null LICs was totally reversed upon Cyclin D2 overexpression (Fig. 3g).

These results indicate that as a direct downstream target of CD274, Cyclin D2 is responsible for AML development. In line with our findings, we also found that Cyclin D2 was significantly upregulated when CD274 was overexpressed in 293T cells (Additional file 6: Figure S5A). The in silico analysis of the curated database or LGA data also showed that the level of expression of Cyclin D2 was much higher in AML cells than in normal hematopoietic stem cells and was inversely correlated with the overall survival of AML patients (Additional file 6: Figure S5B-C) [27].

### CD274 interacts with JNK to increase the Cyclin D2 level

**Fig. 4:**
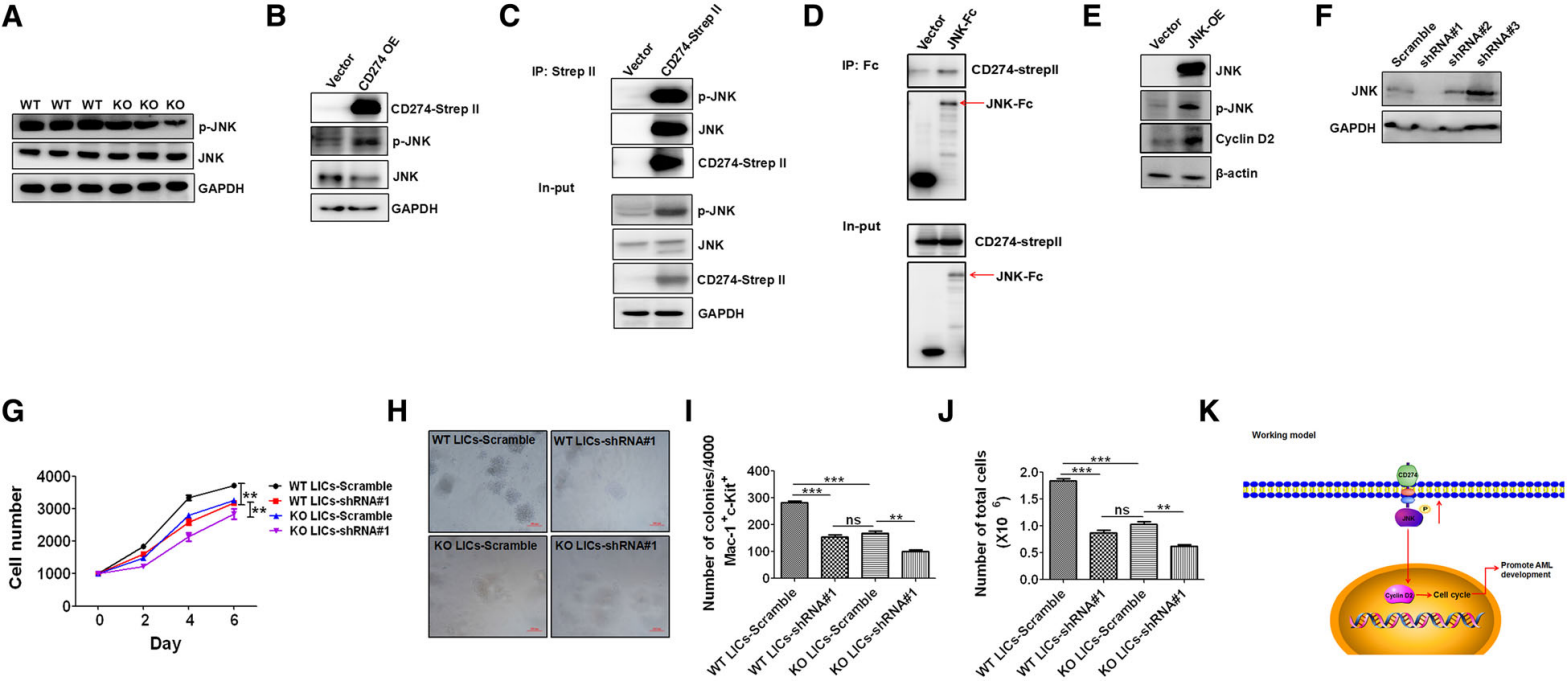
CD274 interacts with JNK to increase Cyclin D2 level. **a** The phospho-JNK and total JNK levels in WT and CD274-null LICs were determined by Western blotting analysis. **b** The phospho-JNK and total JNK levels were evaluated in CD274-over-exprssed (OE) 293T cells by Western blotting analysis. **c** Strep II tagged CD274 was overexpressed in 293T cells, followed by immunoprecipitation with Strep II beads and Western blotting analysis for the levels of CD274, phospho-JNK, and JNK. **d** Fc-tagged JNK was overexpressed in 293T cells, followed by the immunoprecipitation with protein A/G beads and Western blotting analysis for the level of CD274 and JNK. **e** The Cyclin D2 level was evaluated in JNK-overexpressed (OE) 293T cells by Western blotting analysis. **f** The knockdown efficiency of shRNAs (#1-#3) targeting JNK was evaluated in 293 cells by Western blotting analysis. **g** WT and CD274-null LICs were knocked down with shRNA#1 and cultured in solution medium for 6 days in vitro. **h** Representative images for the colony forming units of WT and CD274-null LICs infected with shRNA#1 targeting JNK. **i**, **j** Colony number (**i**) and total cell number of colonies (**j**) in **h** were calculated (*n* = 3, ***P < 0.001, **P < 0.01). **k** Working model for the function of CD274 in leukemogenesis

Because the RNA-sequencing results implicated that MAPK signaling pathway might be involved in the CD274 function in leukemogenesis, and a previous study also suggested that JNK signaling may manipulate the activity of Cyclin D2 [29]. We examined the JNK signaling in WT and CD274-null LICs by Western blotting analysis. As shown in Fig. 4a, the phosphorylation level of JNK was significantly decreased in CD274-null LICs. Consistently, phospho-JNK expression was remarkably elevated upon CD274 overexpression in 293T cells (Fig. 4b). Interestingly, the co-immunoprecipitation experiment revealed that both JNK and phospho-JNK were pulled down by CD274 (Fig. 4c). Meanwhile, CD274 was also detected while JNK was immunoprecipitated (Fig. 4d). Moreover, the expression of Cyclin D2 was significantly upregulated upon JNK overexpression in 293T cells (Fig. 4e). We have further knocked down the expression of JNK in both WT and CD274-null LICs with a shRNA, and the knockdown efficiency was confirmed by Western blotting (Fig. 4f). We demonstrated that the knockdown of JNK caused a dramatic decrease in the proliferation of both WT and CD274-null LICs by solution culture in vitro (Fig. 4g). More importantly, the functional analysis of colony formation units also showed that both the colony and total cell numbers from the JNK-knockdown WT LICs were much fewer compared to the control (Fig. 4g–j). As expected, the knockdown of JNK also led to a less reduction in both the colony and total cell numbers from CD274-null LICs (Fig. 4g–j). In summary, we demonstrate that CD274 directly interacts with JNK to enhance its phosphorylation, which further increases the Cyclin D2 levels to promote cell cycle entry and proliferation (Fig. 4l).

## Discussion

In this study, we evidenced that CD274 plays a critical role in the maintenance of LIC pool, which is independent of its function in the immune checkpoint. We also provided intriguing evidence showing that CD274 directly interplays with JNK and enhances its activities to upregulate Cyclin D2 level, leading to the acceleration of leukemia development. These results consolidate our previous hypothesis that many immune inhibitory molecules are required for the regulation of proliferation of LICs and leukemogenesis apart from their known functions in immune suppression [6].

Previous studies have shown that CD274, which is upregulated in many tumors, functions as a key immune suppressor that hampers the antitumor effect exerted by immune cells, such as the cytotoxic T cells. The treatment with CD274-blocking antibodies was reported to efficiently reduce the tumor burden and restore cytotoxicity activities of CD8^+^ T cells in a murine chronic lymphoblastic leukemia model [30]. Similarly, the results of other investigations also revealed that the overall survival of acute myeloid leukemia mice was dramatically extended upon the treatment with anti-CD274 monoclonal antibodies [31, 32]. In the current study, the expression of CD274 in leukemia cells might have also suppressed the immune responses through binding to its receptor of PD-1 on T cells to enhance the in vivo phenotype due to the use of immunocompetent mice as a recipient to test the leukemogenesis of WT and CD274-null leukemia cells. However, because the recipient mice received lethal irradiation upon transplantation which might have led to the loss of function of immune cells, we believe that these existing immune cells exert only a minor effect on the inhibition of leukemia development, and CD274 has additional functions in the regulation of leukemogenesis.

So far, few studies have been focused on the unraveling of the role of CD274 in the proliferation of LICs. Interestingly, Dong et al. found that the AKT/mTOR pathway was significantly activated in DLBCL cell lines upon a treatment with human recombinant PD1/Fc for 24 h and 48 h [21]. This finding indicates that the ligand engagement may also deliver the reverse signaling to tumor cells themselves apart from the forward signals to suppress the immune response through PD-1, which is consistent with the findings of our study. This is also similar to the function of certain other molecules, such as Ephrins (ligand)/Ephs (receptor). Ephs have the unique capacity to initiate an intercellular signal in both the receptor-bearing cell (“forward” signaling) and the opposing ephrin-bearing cell (“reverse” signaling) following cell-cell contact, which is known as bi-directional signaling [33]. Therefore, we speculate that there also exists a bi-directional signaling upon PD-1/PD-L1 interaction. Consistently, Ishibashi et al. also provide intriguing evidence that CD274 can function as an oncogene to enhance the proliferation and inhibit the apoptosis of myeloma cells [34].

In the current study, we found that CD274 sustains the Cyclin D2 level to promote leukemogenesis. However, how CD274 regulates the expression of Cyclin D2 is yet to be further investigated. The findings of a previous study indicated that doxorubicin can downregulate the expression of CD274 on the surface membrane and promote its nucleus translocation in breast cancer cells [23]. We also found that CD274 exists in both the cytoplasm and the nucleus (data not shown). Nevertheless, CD274 is not associated with Cyclin D2 protein. Our data indicate that JNK signaling may serve as a key mediator between CD274 and Cyclin D2. Interestingly, Song et al. reported that CD274 overexpression promotes cellular proliferation of pancreatic cancer via regulating several cell cycle-related genes and the phosphorylation level of JNK [35]. Herein, we show that CD274 is highly expressed on LICs, which also directly interplay with JNK to maintain its phosphorylation level. We speculate that the intracellular domain of CD274 may be able to recruit certain kinases to further phosphorylate the substrate of JNK. More efforts are required to identify the potential candidate kinase or other cofactors involved in the CD274/JNK/Cyclin D2 signaling.

Moreover, considering that, as reported here, CD274 is localized in the cytoplasm and the nucleus, we speculate that both membranes bound, and the cytoplasmic CD274 may interact with JNK, although this interaction needs to be further clarified. JNK signaling has been found to be involved in the leukemogenesis, and inhibition of JNK pathway may lead to substantial apoptosis in leukemia cells [36–38]. Therefore, understanding how JNK is regulated is critical to develop a novel strategy to target LICs. This study provides some intriguing information for the connection between CD274 and JNK signaling which eventually contributes to a significant upregulation of Cyclin D2 and promotes leukemogenesis. Our results highlight that CD274/JNK/Cyclin D2 signaling controls the cell cycle entry of LICs and leukemia development, which may be helpful for the further identification and understanding of the role of other novel surface immune molecules in the maintenance of LIC pool.

## Conclusions

This study unravels an intriguing role of CD274 in sustaining the proliferation of LICs. CD274 depletion results in a decrease of the expansion ability of LICs. Mechanistically, CD274/JNK/Cyclin D2 signaling enhances the transition from G1 to S phase in the cell cycle of LICs and promotes AML development. Furthermore, CD274 expression level is inversely correlated with the overall survival of AML patients. Our findings shed new light on the treatment for leukemia by targeting certain surface immune molecules of LICs, such as CD274.

## Additional files

Additional file 1: Table S1. Primer sequences for candidate genes. (DOC 34 kb)
Additional file 2: Figure S1. CD274 promotes AML development. (A) Representative flow cytometric plot for the percentages of Mac-1^+^/Gr-1^+^ cells in the recipient mice upon primary transplantation. (B) Quantification results for panel (A) (*n* = 5). (C) Representative flow cytometric plot for the percentages of CD3^+^/B220^+^ cells in the recipient mice upon primary transplantation. (D-E) Representative images and weight of spleens and livers of the mice transplanted with WT or CD274-null leukemia cells upon primary transplantation (*n* = 3). (F) Quantitative results of spleens and livers of the WT or CD274-null leukemic mice upon secondary transplantation (*n* = 3). (G) Representative images for the histological hematoxylin/eosin staining of AML infiltration in the livers and spleens of mice upon secondary transplantation. (PDF 228 kb)
Additional file 3: Figure S2. CD274 is inversely correlated to the overall survival of AML patients. (A) In silico analysis of the expression of CD274 in human AML samples from the curated databases (the HemaExplorer, http://servers.binf.ku.dk/hemaexplorer/). (B) In silico analysis of the relationship between CD274 expression level and overall survival in AML patients from the databases of Leukemia Gene Atlas (LGA) (http://www.leukemia-gene-atlas.org/LGAtlas/). (*, *p* < 0.05). (PDF 176 kb)
Additional file 4: Figure S3. CD274 has no effect on the differentiation and apoptosis of LICs. (A) Representative flow cytometric plot for the percentages of Mac-1^+^/Gr-1^+^ cells in the recipient mice upon secondary transplantation. (B) Quantification results for panel (A) (*n* = 3, *p* > 0.05). (C-E) The apoptotic status was examined by the staining with Annexin V and 7-AAD in WT and CD274-null LICs upon the primary (C-D) or secondary transplantation (E-F) (*n* = 3, *p* > 0.05). (PDF 190 kb)
Additional file 5: Figure S4. Potential downstream targets for CD274. RNA-sequencing was performed with WT and CD274-null LICs, and the candidate genes related to proliferation and cell cycle was analyzed. (PDF 116 kb)
Additional file 6: Figure S5. CD274 promotes the expression of Cyclin D2, which is inversely correlated to the overall survival of AML patients. (A) The expression of Cyclin D2 was measured in CD274-over-expressed (OE) 293T cells by Western blotting analysis. (B-C) In silico analysis for the expression of Cyclin D2 in human AML samples (B, the HemaExplorer, http://servers.binf.ku.dk/hemaexplorer/ ), and for the relationship between Cyclin D2 expression level and overall survival in AML patients (http://www.leukemia-gene-atlas.org/LGAtlas/) (*, *p* < 0.05). (PDF 184 kb)

## Abbreviations

allo-HSCT: Allogeneic hematopoietic stem cell transplantation
AML: Acute myeloid leukemia
FPKM: Fragments per kilo-base per million mapped reads
LICs: Leukemia-initiating cells
LILRB2: Leukocyte immunoglobulin-like receptor subfamily B member 2
YFP: Yellow fluorescent protein

## References

[1] Estey E, Dohner H (2006). Acute myeloid leukaemia. Lancet.

[2] Pulte D, Gondos A, Brenner H (2008). Improvements in survival of adults diagnosed with acute myeloblastic leukemia in the early 21st century. Haematologica.

[3] Middeke JM, Beelen D, Stadler M, Gohring G, Schlegelberger B, Baurmann H, Bug G, Bellos F, Mohr B, Buchholz S, Schwerdtfeger R, Martin H, Hegenbart U, Ehninger G, Bornhäuser M, Schetelig J, Cooperative German Transplant Study Group (2012). Outcome of high-risk acute myeloid leukemia after allogeneic hematopoietic cell transplantation: negative impact of abnl(17p) and -5/5q. Blood.

[4] Paietta E (2012). Minimal residual disease in acute myeloid leukemia: coming of age. Hematology Am Soc Hematol Educ Program.

[5] Kantarjian H, Ravandi F, O'Brien S, Cortes J, Faderl S, Garcia-Manero G, Jabbour E, Wierda W, Kadia T, Pierce S, Shan J, Keating M, Freireich EJ (2010). Intensive chemotherapy does not benefit most older patients (age 70 years or older) with acute myeloid leukemia. Blood.

[6] Zheng J, Umikawa M, Cui C, Li J, Chen X, Zhang C, Huynh H, Kang X, Silvany R, Wan X, Ye J, Cantó AP, Chen SH, Wang HY, Ward ES, Zhang CC (2012). Inhibitory receptors bind ANGPTLs and support blood stem cells and leukaemia development. Nature.

[7] Zhang F, Zheng J, Kang X, Deng M, Lu Z, Kim J, Zhang C (2015). Inhibitory leukocyte immunoglobulin-like receptors in cancer development. Sci China Life Sci.

[8] Jaiswal S, Jamieson CH, Pang WW, Park CY, Chao MP, Majeti R, Traver D, van Rooijen N, Weissman IL (2009). CD47 is upregulated on circulating hematopoietic stem cells and leukemia cells to avoid phagocytosis. Cell.

[9] Frolova O, Benito J, Brooks C, Wang RY, Korchin B, Rowinsky EK, Cortes J, Kantarjian H, Andreeff M, Frankel AE, Konopleva M (2014). SL-401 and SL-501, targeted therapeutics directed at the interleukin-3 receptor, inhibit the growth of leukaemic cells and stem cells in advanced phase chronic myeloid leukaemia. Br J Haematol.

[10] Safaee M, Fakurnejad S, Bloch O, Clark AJ, Ivan ME, Sun MZ, Oh T, Phillips JJ, Parsa AT (2015). Proportional upregulation of CD97 isoforms in glioblastoma and glioblastoma-derived brain tumor initiating cells. PLoS One.

[11] van Pel M, Hagoort H, Hamann J, Fibbe WE (2008). CD97 is differentially expressed on murine hematopoietic stem-and progenitor-cells. Haematologica.

[12] Majeti R, Chao MP, Alizadeh AA, Pang WW, Jaiswal S, Gibbs KD, van Rooijen N, Weissman IL (2009). CD47 is an adverse prognostic factor and therapeutic antibody target on human acute myeloid leukemia stem cells. Cell.

[13] Chao MP, Alizadeh AA, Tang C, Jan M, Weissman-Tsukamoto R, Zhao F, Park CY, Weissman IL, Majeti R (2011). Therapeutic antibody targeting of CD47 eliminates human acute lymphoblastic leukemia. Cancer Res.

[14] Mardiros A, Forman SJ, Budde LE (2015). T cells expressing CD123 chimeric antigen receptors for treatment of acute myeloid leukemia. Curr Opin Hematol.

[15] Agata Y, Kawasaki A, Nishimura H, Ishida Y, Tsubata T, Yagita H, Honjo T (1996). Expression of the PD-1 antigen on the surface of stimulated mouse T and B lymphocytes. Int Immunol.

[16] Chang WS, Kim JY, Kim YJ, Kim YS, Lee JM, Azuma M, Yagita H, Kang CY (2008). Cutting edge. Programmed death-1/programmed death ligand 1 interaction regulates the induction and maintenance of invariant NKT cell anergy. J Immunol.

[17] Crane CA, Panner A, Murray JC, Wilson SP, Xu H, Chen L, Simko JP, Waldman FM, Pieper RO, Parsa AT (2009). PI(3) kinase is associated with a mechanism of immunoresistance in breast and prostate cancer. Oncogene.

[18] Crane C, Panner A, Pieper RO, Arbiser J, Parsa AT (2009). Honokiol-mediated inhibition of PI3K/mTOR pathway: a potential strategy to overcome immunoresistance in glioma, breast, and prostate carcinoma without impacting T cell function. J Immunother.

[19] Concha-Benavente F, Srivastava RM, Trivedi S, Lei Y, Chandran U, Seethala RR, Freeman GJ, Ferris RL (2016). Identification of the cell-intrinsic and -extrinsic pathways downstream of EGFR and IFNgamma that induce PD-L1 expression in head and neck cancer. Cancer Res.

[20] Fujita Y, Yagishita S, Hagiwara K, Yoshioka Y, Kosaka N, Takeshita F, Fujiwara T, Tsuta K, Nokihara H, Tamura T, Asamura H, Kawaishi M, Kuwano K, Ochiya T (2015). The clinical relevance of the miR-197/CKS1B/STAT3-mediated PD-L1 network in chemoresistant non-small-cell lung cancer. Mol Ther.

[21] Dong L, Lv H, Li W, Song Z, Li L, Zhou S, Qiu L, Qian Z, Liu X, Feng L, Meng B, Fu K, Wang X, Pan-Hammarström Q, Wang P, Wang X, Zhang H (2016). Co-expression of PD-L1 and p-AKT is associated with poor prognosis in diffuse large B-cell lymphoma via PD-1/PD-L1 axis activating intracellular AKT/mTOR pathway in tumor cells. Oncotarget.

[22] Schreiner B, Mitsdoerffer M, Kieseier BC, Chen L, Hartung HP, Weller M, Wiendl H (2004). Interferon-beta enhances monocyte and dendritic cell expression of B7-H1 (PD-L1), a strong inhibitor of autologous T-cell activation: relevance for the immune modulatory effect in multiple sclerosis. J Neuroimmunol.

[23] Ghebeh H, Lehe C, Barhoush E, Al-Romaih K, Tulbah A, Al-Alwan M, Hendrayani SF, Manogaran P, Alaiya A, Al-Tweigeri T, Aboussekhra A, Dermime S (2010). Doxorubicin downregulates cell surface B7-H1 expression and upregulates its nuclear expression in breast cancer cells: role of B7-H1 as an anti-apoptotic molecule. Breast Cancer Res.

[24] Krivtsov AV, Twomey D, Feng Z, Stubbs MC, Wang Y, Faber J, Levine JE, Wang J, Hahn WC, Gilliland DG, Golub TR, Armstrong SA (2006). Transformation from committed progenitor to leukaemia stem cell initiated by MLL-AF9. Nature.

[25] Zheng J, Huynh H, Umikawa M, Silvany R, Zhang CC (2011). Angiopoietin-like protein 3 supports the activity of hematopoietic stem cells in the bone marrow niche. Blood.

[26] Zheng J, Umikawa M, Zhang S, Huynh H, Silvany R, Chen BP, Chen L, Zhang CC (2011). Ex vivo expanded hematopoietic stem cells overcome the MHC barrier in allogeneic transplantation. Cell Stem Cell.

[27] Metzeler KH, Hummel M, Bloomfield CD, Spiekermann K, Braess J, Sauerland MC, Heinecke A, Radmacher M, Marcucci G, Whitman SP, Maharry K, Paschka P, Larson RA, Berdel WE, Büchner T, Wörmann B, Mansmann U, Hiddemann W, Bohlander SK, Buske C, Cancer and Leukemia Group B, German AML Cooperative Group (2008). An 86-probe-set gene-expression signature predicts survival in cytogenetically normal acute myeloid leukemia. Blood.

[28] Russo LC, Araujo CB, Iwai LK, Ferro ES, Forti FL. A Cyclin D2-derived peptide acts on specific cell cycle phases by activating ERK1/2 to cause the death of breast cancer cells. J Proteomics. 2016;doi:10.1016/j.jprot.2016.06.028. 10.1016/j.jprot.2016.06.028 27371349

[29] Turchi L, Loubat A, Rochet N, Rossi B, Ponzio G (2000). Evidence for a direct correlation between c-Jun NH2 terminal kinase 1 activation, cyclin D2 expression, and G(1)/S phase transition in the murine hybridoma 7TD1 cells. Exp Cell Res.

[30] McClanahan F, Hanna B, Miller S, Clear AJ, Lichter P, Gribben JG, Seiffert M (2015). PD-L1 checkpoint blockade prevents immune dysfunction and leukemia development in a mouse model of chronic lymphocytic leukemia. Blood.

[31] Zhou Q, Munger ME, Highfill SL, Tolar J, Weigel BJ, Riddle M, Sharpe AH, Vallera DA, Azuma M, Levine BL, June CH, Murphy WJ, Munn DH, Blazar BR (2010). Program death-1 signaling and regulatory T cells collaborate to resist the function of adoptively transferred cytotoxic T lymphocytes in advanced acute myeloid leukemia. Blood.

[32] Zhang L, Gajewski TF, Kline J (2009). PD-1/PD-L1 interactions inhibit antitumor immune responses in a murine acute myeloid leukemia model. Blood.

[33] Daar IO (2012). Non-SH2/PDZ reverse signaling by ephrins. Semin Cell Dev Biol.

[34] Ishibashi M, Tamura H, Sunakawa M, Kondo-Onodera A, Okuyama N, Hamada Y, Moriya K, Choi I, Tamada K, Inokuchi K (2016). Myeloma drug resistance induced by binding of myeloma B7-H1 (PD-L1) to PD-1. Cancer Immunol Res.

[35] Song X, Liu J, Lu Y, Jin H, Huang D (2014). Overexpression of B7-H1 correlates with malignant cell proliferation in pancreatic cancer. Oncol Rep.

[36] Hsiao PC, Hsieh YH, Chow JM, Yang SF, Hsiao M, Hua KT, Lin CH, Chen HY, Chien MH (2013). Hispolon induces apoptosis through JNK1/2-mediated activation of a caspase-8, -9, and -3-dependent pathway in acute myeloid leukemia (AML) cells and inhibits AML xenograft tumor growth in vivo. J Agric Food Chem.

[37] Zhao Q, Assimopoulou AN, Klauck SM, Damianakos H, Chinou I, Kretschmer N, Rios JL, Papageorgiou VP, Bauer R, Efferth T (2015). Inhibition of c-MYC with involvement of ERK/JNK/MAPK and AKT pathways as a novel mechanism for shikonin and its derivatives in killing leukemia cells. Oncotarget.

[38] Calvino E, Tejedor MC, Sancho P, Herraez A, Diez JC (2015). JNK and NFkappaB dependence of apoptosis induced by vinblastine in human acute promyelocytic leukaemia cells. Cell Biochem Funct.

