# Supplementary material for: CD274 promotes cell cycle entry of leukemia-initiating cells through JNK/Cyclin D2 signaling
Source: J Hematol Oncol. 2016 Nov 17;9:124. doi: 10.1186/s13045-016-0350-6 (PMC5114730; doi:10.1186/s13045-016-0350-6)
Supplement: Additional file 1: Table S1. — Primer sequences for candidate genes. (DOC 34 kb) [file 13045_2016_350_MOESM1_ESM.doc]

**Additional file 1: Table S1. Primer Sequences for candidate genes**

| **Gene** | **Sequence (target sequence)** |
| --- | --- |
| mouse CD274-F | GCTCCAAAGGACTTGTACGTG |
| mouse CD274-R | TGATCTGAAGGGCAGCATTTC |
| Mouse Cyclin D2-F | GAGTGGGAACTGGTAGTGTTG |
| Mouse Cyclin D2-R | CGCACAGAGCGATGAAGGT |
| Mouse p21-F | GGGCGCACGATGTTCAGAA |
| Mouse p21-R | CACCACCAGGTCGAAATGGG |
| Mouse p16-F | ATGCTCCCGTCTTTGCAGG |
| Mouse p16-R | CATCACTATCCCCTCGATTCTGT |
| Mouse p53-F | TCACAGCGTCTGTTGACATTT |
| Mouse p53-R | ACCAAGCTCATTACCCTGACA |
| Mouse CDK6-F | GGCGTACCCACAGAAACCATA |
| Mouse CDK6-R | AGGTAAGGGCCATCTGAAAACT |
| Mouse β-actin-F | ATGACCCAAGCCGAGAAGG |
| Mouse β-actin-R | CGGCCAAGTCTTAGAGTTGTTG |
| Mouse JNK-shRNA#1 | GCAAGAGATTTGTTATCCAAA |
