# Supplementary figures and images for: CD274 promotes cell cycle entry of leukemia-initiating cells through JNK/Cyclin D2 signaling
Source: J Hematol Oncol. 2016 Nov 17;9:124. doi: 10.1186/s13045-016-0350-6 (PMC5114730; doi:10.1186/s13045-016-0350-6)

**A**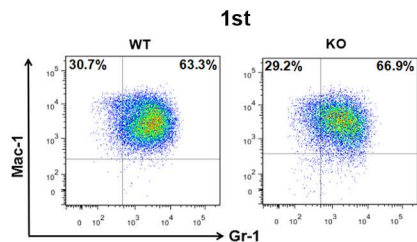**B**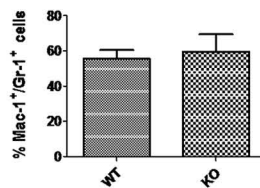**D**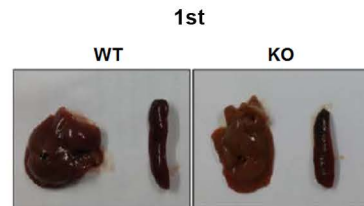**C**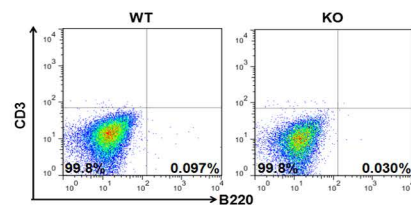**E**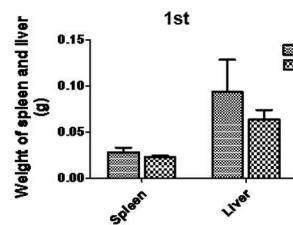**F**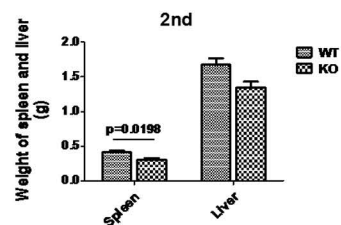**G**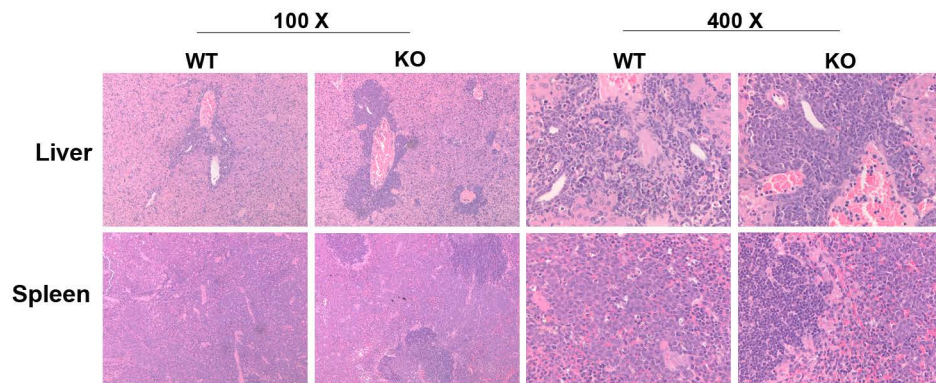

Supplement: Additional file 2: Figure S1. — CD274 promotes AML development. (A) Representative flow cytometric plot for the percentages of Mac-1+/Gr-1+ cells in the recipient mice upon primary transplantation. (B) Quantification results for panel (A) (n = 5). (C) Representative flow cytometric plot for the percentages of CD3+/B220+ cells in the recipient mice upon primary transplantation. (D-E) Representative images and weight of spleens and livers of the mice transplanted with WT or CD274-null leukemia cells upon primary transplantation (n = 3). (F) Quantitative results of spleens and livers of the WT or CD274-null leukemic mice upon secondary transplantation (n = 3). (G) Representative images for the histological hematoxylin/eosin staining of AML infiltration in the livers and spleens of mice upon secondary transplantation. (PDF 228 kb) [file 13045_2016_350_MOESM2_ESM.pdf]

A

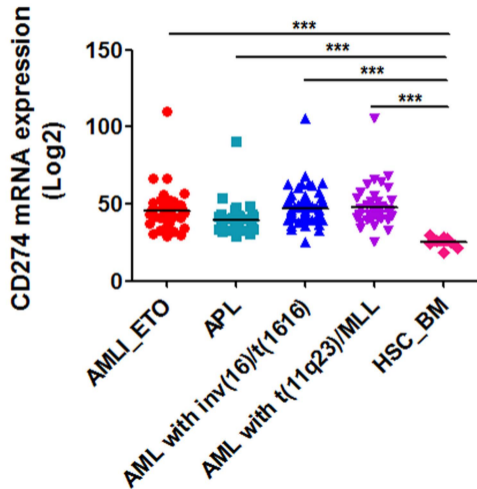

B

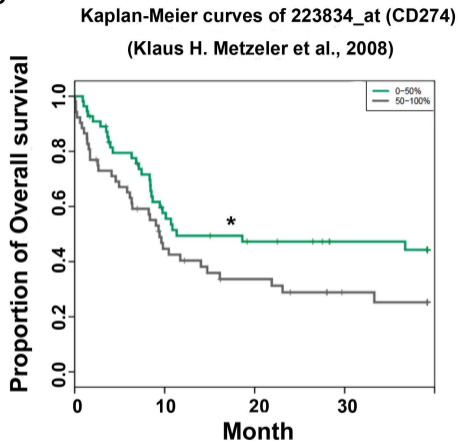

Supplement: Additional file 3: Figure S2. — CD274 is inversely correlated to the overall survival of AML patients. (A) In silico analysis of the expression of CD274 in human AML samples from the curated databases (the HemaExplorer, http://servers.binf.ku.dk/hemaexplorer/). (B) In silico analysis of the relationship between CD274 expression level and overall survival in AML patients from the databases of Leukemia Gene Atlas (LGA) (http://www.leukemia-gene-atlas.org/LGAtlas/). (*, p < 0.05). (PDF 176 kb) [file 13045_2016_350_MOESM3_ESM.pdf]

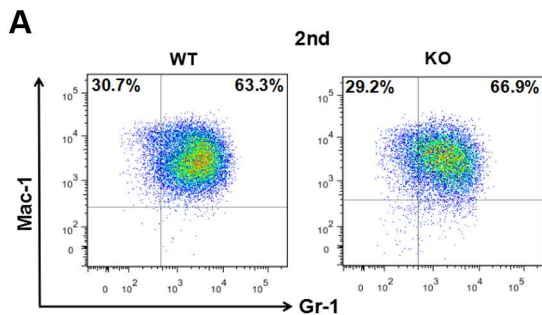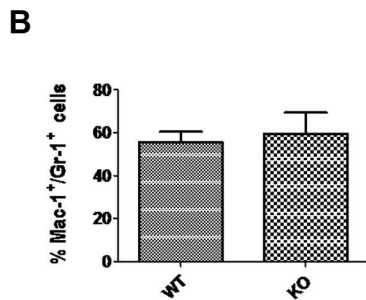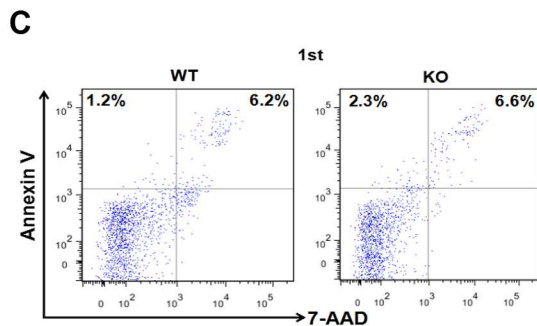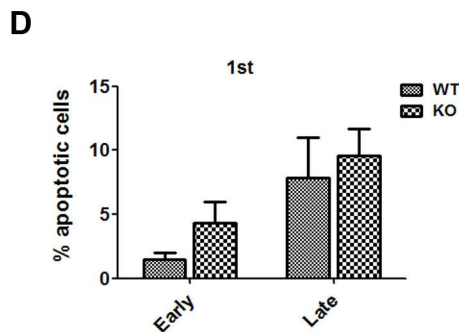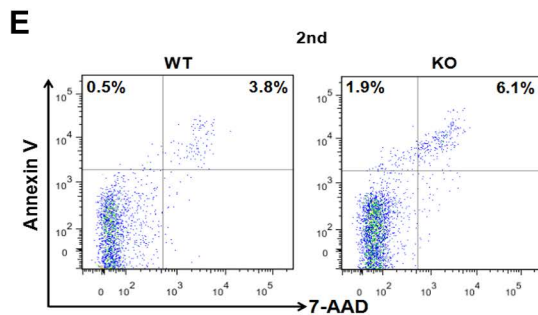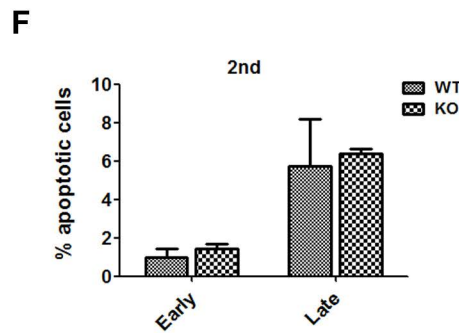

Supplement: Additional file 4: Figure S3. — CD274 has no effect on the differentiation and apoptosis of LICs. (A) Representative flow cytometric plot for the percentages of Mac-1+/Gr-1+ cells in the recipient mice upon secondary transplantation. (B) Quantification results for panel (A) (n = 3, p > 0.05). (C-E) The apoptotic status was examined by the staining with Annexin V and 7-AAD in WT and CD274-null LICs upon the primary (C-D) or secondary transplantation (E-F) (n = 3, p > 0.05). (PDF 190 kb) [file 13045_2016_350_MOESM4_ESM.pdf]

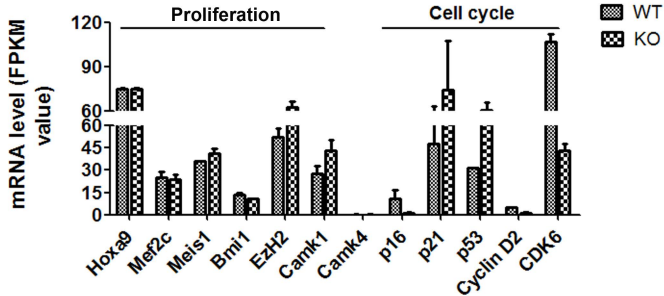

Supplement: Additional file 5: Figure S4. — Potential downstream targets for CD274. RNA-sequencing was performed with WT and CD274-null LICs, and the candidate genes related to proliferation and cell cycle was analyzed. (PDF 116 kb) [file 13045_2016_350_MOESM5_ESM.pdf]

**A**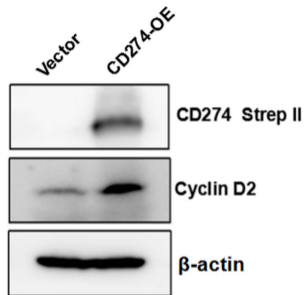**B**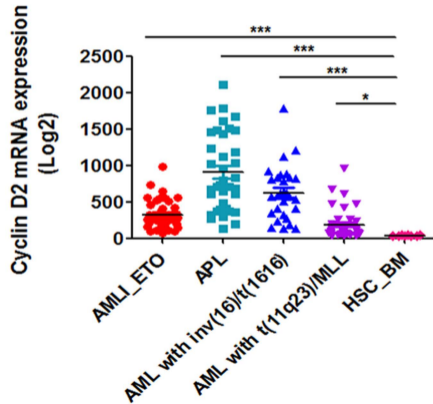**C**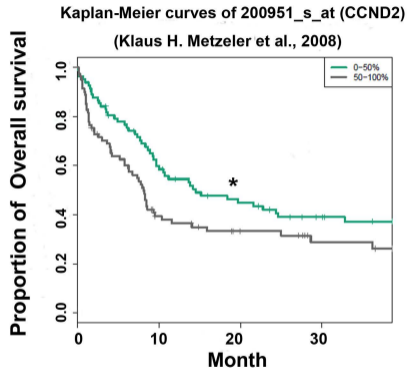

Supplement: Additional file 6: Figure S5. — CD274 promotes the expression of Cyclin D2, which is inversely correlated to the overall survival of AML patients. (A) The expression of Cyclin D2 was measured in CD274-over-expressed (OE) 293T cells by Western blotting analysis. (B-C) In silico analysis for the expression of Cyclin D2 in human AML samples (B, the HemaExplorer, http://servers.binf.ku.dk/hemaexplorer/ ), and for the relationship between Cyclin D2 expression level and overall survival in AML patients (http://www.leukemia-gene-atlas.org/LGAtlas/) (*, p < 0.05). (PDF 184 kb) [file 13045_2016_350_MOESM6_ESM.pdf]
